# Novel biochemical predictors of unfavorable prognosis for stable coronary disease

**DOI:** 10.1097/MD.0000000000012372

**Published:** 2018-09-14

**Authors:** Andrzej Cacko, Agnieszka Kondracka, Monika Gawałko, Renata Główczyńska, Krzysztof J. Filipiak, Zbigniew Bartoszewicz, Grzegorz Opolski, Marcin Grabowski

**Affiliations:** a 1st Department of Cardiology; b Department of Medical Informatics and Telemedicine; c Department of Internal Diseases and Endocrinology, Medical University of Warsaw, Warsaw, Poland.

**Keywords:** acute coronary syndrome, biomarkers, risk stratification

## Abstract

Successful risk stratification is necessary for optimum management of patients after acute coronary syndrome (ACS). The aim of the study was to evaluate the role of novel biochemical markers in the prediction of adverse cardiovascular events in stable patients several years after ACS.

The study group was randomly selected from all ACS patients treated with reperfusion therapy between 2002 and 2003 at 1st Department of Cardiology, Medical University of Warsaw, Poland. All patients were readmitted to hospital between 2010 and 2011 for clinical and biochemical cardiovascular risk factors assessment and were prospectively observed for 30-months follow-up. The primary endpoint was all-cause death or hospital readmissions due to a cardiovascular condition at 30 months. The secondary endpoint was a composite of all-cause death or hospitalization-related noncardiovascular condition during the follow-up.

The study population consisted of 146 patients (mean age 66.6 ± 9.8 years; 60 female). The primary and secondary endpoints occurred in 49 and 65 patients, respectively. Univariate analysis demonstrated that out of 17 analyzed biomarkers only high-sensitive C-reactive protein (hsCRP), Soluble Fms-Like Tyrosine kinase-1 (sFlt-1), and endothelin-1 (ET-1) were significantly associated with primary end-point and N-Terminal pro-B-type natriuretic peptide (NT-proBNP), hsCRP, ET-1, sFlt-1, and procalcitonin (PCT)—with secondary end-point. Multivariate analysis demonstrated that concentration of sFlt-1 was the only independent factor associated with primary end-point (*P* = .007 and *P* = .025, respectively), whereas NT-proBNP and hsCRP levels were only associated with secondary end-point (*P* = .004 and *P* = .001, respectively).

sFlt-1, NT-proBNP, and hsCRP are associated with adverse outcomes in stable patients several years after ACS and may emerge as useful clinical biomarkers to enhance stratify patient's risk.

## Abstract 2

Supplemental Digital Content is available in the text

## 1 Introduction

Readmission following an acute coronary syndrome (ACS) is frequent in our community and up to 30% of discharged patients need rehospitalization within 6 months.^[1]^ Moreover, the overall 1 and 3-year mortality of ACS patients after hospital discharge ranged from 19.4% to 28.2% respectively.^[2]^ Thus further risk stratification is mandatory to avoid needless hospitalizations and to prevent deaths caused by complications of ACS.^[3–6]^ Several ACS prognostic risk scores are available, but most of them have been validated with respect to in-hospital and short-term (30-day) use.^[7–10]^

Risk factors for long-term prognosis of ACS survivors and of the population with stable coronary artery disease are similar. Adverse clinical events have been positively correlated with the presence of angina, concomitant diseases, heart failure symptoms, and impaired left ventricular systolic function.^[4,11–13]^ However, predictive value of novel biochemical risk factors analyzed in stable coronary artery disease is not verified in the population of patients who survived several years after ACS.

The aim of the study was to evaluate the role of novel biochemical markers in the prediction of adverse cardiovascular events in stable patients several years after ACS treated with reperfusion therapy.

## 2 Methods

### 2.1 Study population

Out of a total of 650 patients hospitalized for ACS and treated with reperfusion therapy between 2002 and 2003 at 1st Department of Cardiology, Medical University of Warsaw, Poland, 150 patients with nonfatal ACS who survived the 7 years after discharge were randomly selected to participate in our study, mainly based on shortest distance between patient residence and hospital location. Four patients declined research participation. All final analytic cohort of 146 patients were readmitted to hospital between November 2010 and October 2011 for clinical and biochemical cardiovascular risk factors assessment and were prospectively observed for a mean follow-up period of 30 months. The ethical approval before initiation of the study was obtained. All patients signed the informed consent before inclusion into the study.

Collected patients’ data regarding demographic characteristics, medical history, cardiovascular risk factors, clinical presentation, reperfusion therapy, laboratory tests, hospital length of stay, and discharge medications were retrospective obtained from medical records concerning first patient's admission due to ACS. The evaluation after 7 years of ACS involved clinical presentation (severity of angina pectoris and heart failure symptoms), laboratory tests (renal function and lipid profile), and echocardiographic examination.

### 2.2 Biomarker assessment

In all patients included in the prospective observation blood samples were taken to establish the levels of following novel biochemical risk factors:

1. Inflammatory markers, that is, growth, cell migration, and angiogenesis factors (high-sensitive C-reactive protein (hsCRP), interleukin-6 (IL-6), procalcitonin (PCT), tumor necrosis factor alpha (TNF-alpha), Matrix Metaloproteinas-9 (MMP-9), endothelin-1 (ET-1), Pregnancy-Associated Plasma Protein-A (PAPP-A), Soluble Fms-Like Tyrosine kinase-1 (sFlt-1), Neutrophil Gelatinase-Associated Lipocalin (NGAL), Platelet-Derived Growth Factor-AA (PDGFAA), Procollagen type II N-terminal propeptide (PIIANP), Placental Growth Factor).
2. Markers of hemodynamic overload of the myocardium (N-Terminal pro-B-type natriuretic peptide (NT-proBNP)), ischemia/myocardial necrosis markers (high-sensitive cardiac troponin-I (hsTnI), ischemia-modified albumin (IMA)).
3. Others (vitamin-D (VIT-D) and aldosterone).

Peripheral venous blood samples were taken without anticoagulants from all participants after overnight fast. Blood samples were centrifuged at 1000 g for 15 minutes and the sera/plasma were aliquoted and stored at −80°C until analysis. Manual EIA kits were used to measure: aldosterone, TNF-alfa, MMP-9, ET-1, PIIANP (DRG International, Mountainside, NJ), PDGFAA (R&D Systems, Minneapolis, MN), NGAL (BioPorto Diagnostics A/S, Gentofte, Denmark) and IMA (USCN Life Science, Wuhan, China). Roche Diagnostics laboratory kits were used to conduct NT-pro BNP, hsCRP, SFLT-1, hsTnI, placental growth factor, PAPP-A, IL-6, VIT-D, and PCT tests using Cobas 6000 analyzer.

Description of test used for biomarkers’ analysis (names, detections limits, inter-, and intra-assay variation, costs) is included in Supplemental Content (Table S1).

### 2.3 Transthoracic echocardiography

Resting transthoracic echocardiography (TTE) using EPIQ 7 or iE33 Ultrasound Machine (Philips Medical Systems, Andover, MA) was performed to asses left ventricular end-diastolic volume, left ventricle ejection fractal using modified biplane Simpson method from the apical 2 and 4 chamber view, left ventricular mass was derived from M-mode echocardiography.

### 2.4 Clinical endpoints at 30-months follow-up

The follow-up data were collected by personal visits or telephonic contact (if the patient was unable to attend the ambulatory visit). In case of receiving information about patient's death, it was verified based on data obtained from the National Death Registry of Poland.

The primary endpoint was all-cause death or hospital readmissions due to a cardiovascular condition during 30 months. The secondary endpoint was a composite of all-cause death or hospitalization related to any reason during the follow-up.

Cardiovascular hospitalization includes hospitalization due to the ACS, heart failure, stroke, intracerebral hemorrhage, cardiovascular procedures, and hospitalization due to other cardiovascular causes, as previously described.^[14]^

### 2.5 Statistical analysis

Continuous variables were presented as mean ± standard deviation and categorical variables are presented as percentages. Mann–Whitney test was used to compare continuous variables (novel biochemical factors). To determine whether any novel biochemical factors were related to primary or secondary endpoint, univariate and multivariate logistic regressions were performed. A multivariate logistic regression analysis was performed using clinical variables with a *P* value of.10 or less in a univariate analysis. Cox proportional hazards regression models were used to estimate hazard ratios (HRs) and their 95% confidence intervals (CIs). Statistical analyses were performed by using the using MedCalc Statistical Software version 14.12.0 (MedCalc Software, Ostend, Belgium). For all analyses, a *P* value of less than.05 was considered statistically significant.

## 3 Results

The study population consisted of 146 patients (mean age 66.6 ± 9.8 years; 60 (41%) female). Detailed characteristics collected at admission for ACS is presented in Supplemental Content (Table S2) and characteristics collected at the beginning of prospective observation is presented in Table 1. Patients’ characteristic, demographic data, and medical prognosis were also presented in the paper published recently in indexed journal.^[15]^ Follow-up was completed for every patient. Over a mean follow-up time of 30 months, 16 (11%) patients died. Primary and secondary endpoints occurred in 49 (33.5%) and 65 (44.5%) patients, respectively.

**Table 1:**
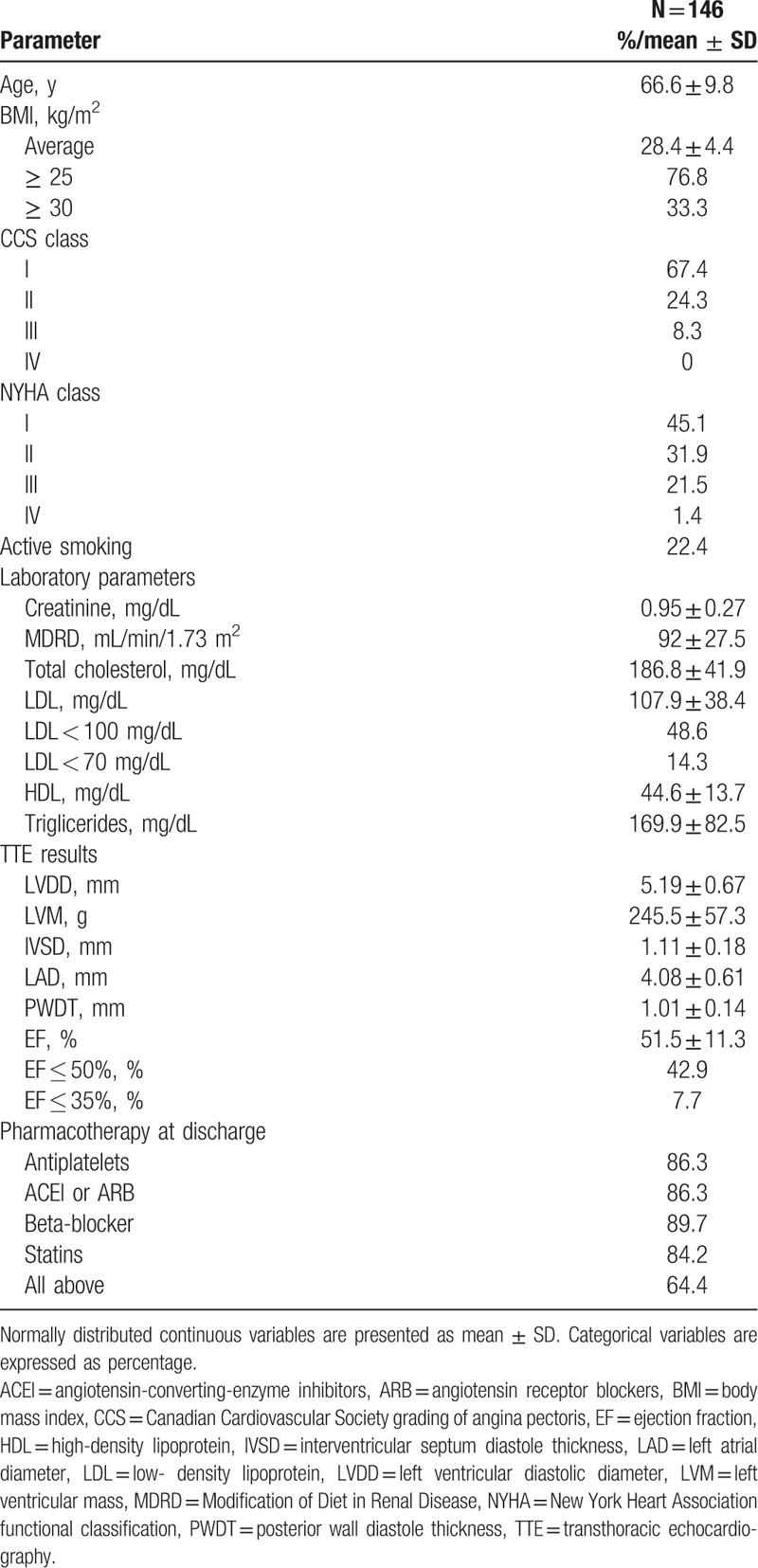
Baseline characteristics at the beginning of prospective observation.

Mean concentrations of novel biochemical cardiovascular markers are presented in Table 2. The primary outcome occurred more frequently in patients with significantly higher levels of NT-proBNP (*P* = .025) and sFlt-1 (*P* = .015) and with lower levels of IL-6 (*P* = .042) compared with other patients. The concentration of hsCRP was higher in patients who met the primary endpoint, but did not reach statistical significance (*P* = .064). Concentrations of NT-proBNP (*P* < .001), sFlt-1 (*P* = 009), and hsCRP (*P* = .02) were significantly higher and concentrations of procollagen type II N-terminal propeptide (*P* = .032) and IL-6 (*P* = .014) were significantly lower among patients who met secondary endpoint compared with other patients. The results are shown in Table 3.

**Table 2:**
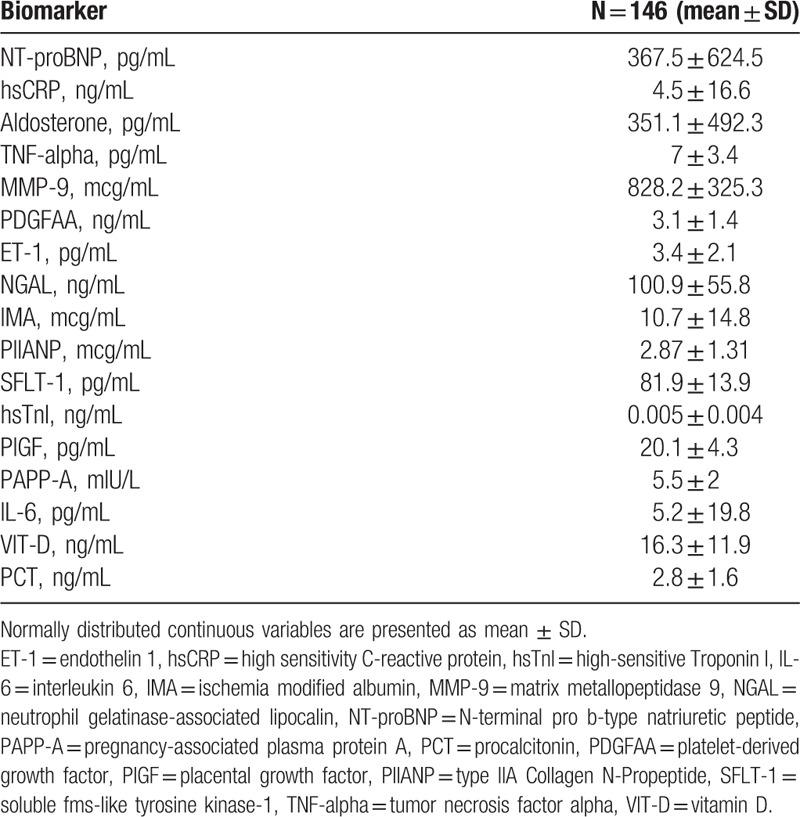
Mean novel biomarkers concentration for cardiovascular risk prediction.

**Table 3:**
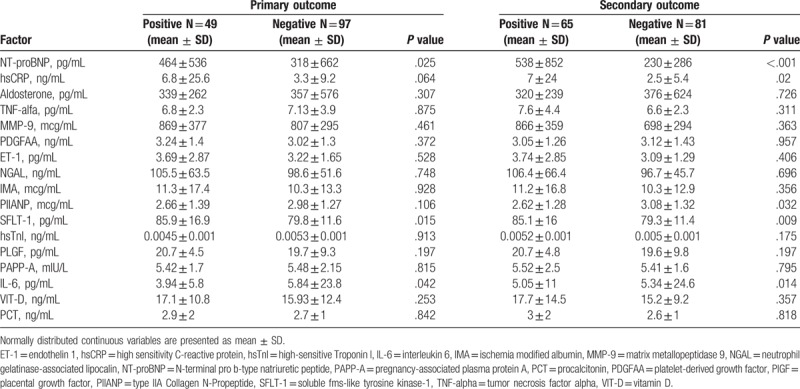
Mean concentrations of novel biomarkers for cardiovascular risk among patients with and without primary and secondary endpoint.

Concentrations of hsCRP (*P* = .017), SFLT-1 (*P* = .003), and ET-1 (*P* = .021) significantly influenced the primary outcome in univariate Cox proportional hazard regression model. Influence of concentrations of NT-proBNP and PCT approached, but did not reach, significance for primary endpoint (*P* = .098 and *P* = .066, respectively). In similar univariate Cox proportional hazard regression model the risk of secondary outcome depended on concentrations of NT-proBNP (*P* = .001), hsCRP (*P* < .001), ET-1 (*P* = .002), SFLT-1 (*P* = .003), and PCT (*P* = .004). Results of statistical analysis are presented in Table 4.

**Table 4:**
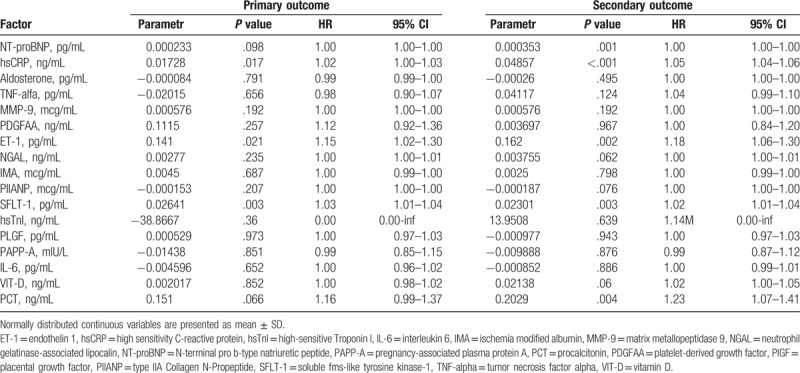
Univariate statistical analysis of novel biomarkers for a prediction of primary and secondary endpoint.

Multivariate analysis demonstrated that concentration of sFlt-1 was the only independent factor associated with the primary and secondary endpoints (*P* = .007 and *P* = .025, respectively), whereas NT-proBNP and hsCRP levels were only associated with secondary endpoint (*P* = .004 and *P* = .001), respectively). Results of multivariate statistical analysis are presented in Table 5.

**Table 5:**
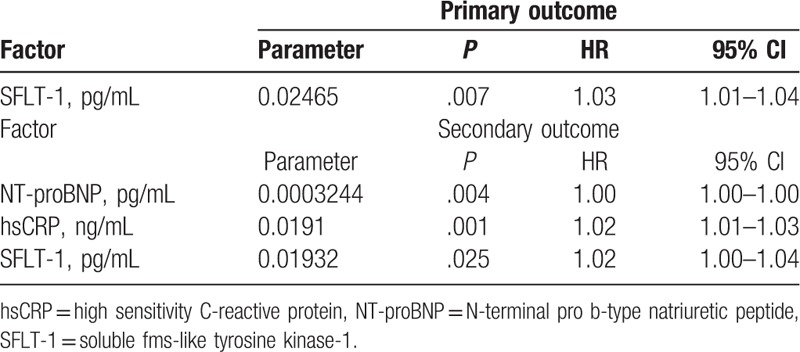
Multivariate statistical analysis of novel biomarkers for a primary and secondary endpoint.

## 4 Discussion

The present study was aimed at simultaneously analysis of 17 biochemical markers described in the clinical literature as cardiovascular risk factors. To the best of our knowledge, this is the first report describing such a wide spectrum of biochemical markers in prospective cohort study that comprised clinically stable patients several years after hospitalization due to ACS. We would like to emphasize the importance of this report with regard to ensuring better cardiovascular risk prediction and prevention among survivors of ACS.

The major finding of this study was that elevated sFlt-1 concentration was strongly associated with higher all-cause death and hospitalization risk. Consistent with our findings, studies of sFlt-1 in other cardiovascular diseases also have associated elevated sFlt-1 with worse outcomes. Onoue et al reported in a study that included 174 patients with ACS who subsequently develop heart failure, circulating levels of sFlt-1 were significantly higher in patients who developed severe compared with those with stable hemodynamics (611.4 ± 373.6 vs 494.6 ± 243.9 pg/mL, *P* = .016). Moreover, circulating levels of sFlt-1 on admission were directly related to duration of hospitalization.^[16]^ In Weber et al study of 1136 consecutive ACS patients, sFlt-1 levels measured at day 1 of patients who died during follow-up were significantly higher as compared with patients who survived (215 vs 96 pg/mL; *P* < .001). In addition, the mortality rate increased with increasing quartiles of sFlt-1 values (1.0%, 1.0%, 4.9%, and 13.6%).^[17]^ Another research pointed to the importance of this marker comprised group of patients with chronic heart failure, where sFlt-1 concentration was independently associated with measures of heart failure severity, including New York Heart Association Class (*P* < .01). Moreover, in the same study, patients with level of sFlt-1 > 379 pg/mL had an over 6-fold increased risk of adverse outcomes (*P* < .01).^[18]^ It is worth noting that all mentioned studies present results with ACS survivors and an average follow-up of 2 years, what makes our work unique by describing prognostic value of sFlt-1 concentration almost 7 years after ACS and with 2.5-year follow-up.

Another biochemical factor that was significantly associated with the primary and secondary endpoint in our analysis was the hsCRP concentration. It was 2 times higher in patients who met primary endpoint and almost 3 times higher in those who met secondary endpoint. CRP is one of the best-studied acute-phase proteins. Its measurements are currently performed with a high sensitivity method, enabling us to identify patients with increased cardiovascular risk.^[19–22]^ CRP is an independent cardiovascular risk factor in the population of patients with coronary artery disease. Of the 12 markers measured, hs-CRP was the strongest univariate predictor of the risk of cardiovascular events among 28,263 apparently healthy postmenopausal women over a mean follow-up period of 3 years.^[23]^ Another trial outlining the role of hsCRP in contributing to increased cardiovascular risk, was Justification for the Use of Statins in Prevention: an Intervention Trial Evaluating Rosuvastatin study. The researchers showed that therapeutic intervention (administration of rosuvastatin) in subjects without previously diagnosed cardiovascular disease and with normal low-density lipoprotein concentration but with hsCRP concentration > = 2 mg/L during a mean observation period of 2 years reduced general mortality by 20%.^[24]^

Another independent predictor of the secondary endpoint was NT-proBNP. It is worth noting that more than half of study group (54.9%) had symptoms of heart failure (at least in NYHA class II) at the start of prospective observation. The role of the natriuretic peptides in the diagnosis and risk stratification of patients with chronic heart failure is well documented. BNP and NT-proBNP are biomarkers listed in up-to-date European Cardiology Society recommendations for heart failure management as well as evaluated in the population of patients with ACS history.^[25–27]^ The relationship between the concentration of NT-proBNP and the risk for hospitalization and mortality is curvilinear in patients with ACS. Several possible mechanisms can explain the prognostic value of NT-proBNP concentration in the general population. First, higher NT-proBNP concentration may reflect the presence of structural heart disease or cardiac remodeling resulting from increased cardiac stretch.^[28]^ Second, elevated NT-proBNP concentration correlates with the degree of systemic atherosclerosis.^[29]^ In our study NT-proBNP concentration was significantly higher among patients with cardiovascular events. However, it was not included into the regression model in the multivariate analysis in our study. At this point we may consider it as a result of patients’ stable condition. It should be pointed that NT-proBNP concentration varies significantly in time as it changes with a modification in lifestyle or medication during the follow-up.^[30]^ Moreover, overweight and obese adults had lower NT-proBNP concentrations than those in the normal weight.^[31,32]^ There was a paradoxical association between obesity and prognosis in patients with heart failure.^[33]^ The inverse relationship between the NT-proBNP concentrations and body mass index might be explained by an increase in the degradation of the adipose tissue peptide.^[34]^ In our study 76.8% of patients were overweight and 33.3% were obese. Therefore, it could also partially explain the lack of predictive value of NT-proBNP for primary end-point as World Health Organization indices obesity among the most important behavioral risk factors of cardiovascular disease and stroke.^[35]^

The use of ET-1 and PCT in risk prediction in the studied population remains an interesting topic. These biochemical markers showed high predictive value in the univariate analysis, but not in multivariate analysis. ET-1 is a powerful vasoconstrictor predominantly produced by the endothelial cell, with inflammatory action (promote IL-6 release from small airway epithelial cells).^[36,37]^ On the other hand, the presence of inflammatory mediators stimulates the ET-1 secretion.^[38,39]^ Recent studies also showed a positive correlation between ET-1 and other inflammatory mediators including CRP and TNF-alfa, as well as NT-proBNP concentrations in patients with chronic heart failure.^[40,41]^

PCT concentration in patients with chronic heart failure or stable coronary artery disease is elevated regardless of infections.^[42–44]^ Erren et al reported that increased PCT concentrations are correlated with the extent of atherosclerosis in patients with coronary artery disease and peripheral arterial disease.^[45]^ In Sinning et al study, the authors hypothesized that elevated PCT concentration is a reason of a nonspecific cytokine release and an indicator of local ischemic myocardium damage due to coronary artery disease. They found that concentration of PCT increased stepwise according to the number of affected coronary arteries.^[43]^ On the other hand, Rogler and Rosano correlate higher PCT concentration with a disturbed intestinal barrier due to dysfunction of a microcirculation. As chronic heart failure disturb intestinal barrier it may induce a translocation of bacteria and their products and trigger the increase of a PCT concentration.^[45]^ Thus, Sandek et al^[46]^ reported an increased wall thickness of both small and large intestines and larger amounts of adherent bacteria within mucus in chronic heart failure patients.

Interestingly, most of patients that reached the primary and secondary end points were in the NYHA Class I or II. One appealing explanation for these results is that the acute condition responsible for the index admission weakens the overall health of the patient and induces a higher risk of complications or exacerbations related to the (previously) stable comorbidity. Moreover, it is worth noting that cardiovascular or all-cause hospitalization during the follow-up focused attention not just on the primary index admission diagnosis but also on the other comorbidities that patients had.

In this context, the value of screened biomarkers adds to prediction over simple clinical information. However, it is worth remembering that a statistically significant association between the biomarker and the outcome, even in a multivariable model, is not sufficient to determine predictive value. Odds ratios or hazards ratios can be statistically significant even if there is a large overlap in the biomarker distributions of those who do and do not develop disease. Thus, for any given value of a biomarker, there could be an appreciable probability that the individual is a member of either group, diminishing the predictive value of the biomarker.

The relatively low cost of hsCRP is worth noting (twice cheaper than the less available biomarkers), particularly it was an independent factor associated with secondary end-point. Moreover, the cost of sFlt-1 that was associated with primary end-point was the same as NT-proBNP (one of the most common laboratory tests) and may prompt the introduction of the sFlt-1 testing in everyday practice.

### 4.1 Limitations of the study

There are some limitations of the study. First, the study population was relatively small as it was a single-center study. However, there are also advantages to a single-center location, including the possibility of following all subjects closely for the duration of the study and gathering considerably detailed information on each study participant. The size of studied population is a result of applied methodology, that is, enrollment of patients included into the ACS register 7 years before. The costs associated with numerous reagents also influenced the size of the study group and determined the absent of the control group. Thus, it is worth mentioning that a wide range of analyzed biochemical factors enabled discrimination of certain markers potentially significant in future analyses.

The next limitation is lack of data regarding the cause of death. Nevertheless, it must be emphasized that the authors dispose of an accurate analysis of patients’ date of death (from the National Death Registry of Poland) and causes of hospitalization.

## 5 Conclusion

In conclusion, sFlt-1, NT-proBNP, and hsCRP concentrations are associated with adverse outcomes in stable patients several years after ACS and may emerge as useful clinical biomarkers to enhance patient risk stratification beyond currently used approaches.

## Author contributions

**Conceptualization:** Andrzej Cacko, Agnieszka Kondracka, Monika Gawałko, Renata Główczyńska, Krzysztof J Filipiak, Zbigniew Bartoszewicz, Grzegorz Opolski, Marcin Grabowski.

**Data curation:** Andrzej Cacko, Agnieszka Kondracka, Monika Gawałko, Renata Główczyńska, Krzysztof J Filipiak, Zbigniew Bartoszewicz, Grzegorz Opolski, Marcin Grabowski.

**Formal analysis:** Andrzej Cacko, Agnieszka Kondracka, Monika Gawałko, Renata Główczyńska, Krzysztof J Filipiak, Zbigniew Bartoszewicz, Grzegorz Opolski, Marcin Grabowski.

**Methodology:** Andrzej Cacko, Agnieszka Kondracka, Monika Gawałko, Renata Główczyńska, Krzysztof J Filipiak, Zbigniew Bartoszewicz, Grzegorz Opolski, Marcin Grabowski.

**Writing – original draft:** Andrzej Cacko, Agnieszka Kondracka, Monika Gawałko, Renata Główczyńska, Krzysztof J Filipiak, Zbigniew Bartoszewicz, Grzegorz Opolski, Marcin Grabowski.

**Writing – review & editing:** Andrzej Cacko, Agnieszka Kondracka, Monika Gawałko, Renata Główczyńska, Krzysztof J Filipiak, Zbigniew Bartoszewicz, Grzegorz Opolski, Marcin Grabowski.

## Supplementary Material

Supplemental Digital Content
