# Supplementary material for: Novel biochemical predictors of unfavorable prognosis for stable coronary disease
Source: Medicine (Baltimore). 2018 Sep 14;97(37):e12372. doi: 10.1097/MD.0000000000012372 (PMC6155940; doi:10.1097/MD.0000000000012372)
Supplement: Supplemental Digital Content [file medi-97-e12372-s001.doc]

Table S1. Description of test used for biomarkers' analysis

| Test name | sample | LoD  (low limit of detection) | intraassay variation  (precision)  (%) | interassay variation  (repeatability)  (%) | Cost, netto/90 samples (EUR) |
| --- | --- | --- | --- | --- | --- |
| aldosterone, DRG International | serum | 14 pg/mL | 3,2-6,9 | 6,0-8,2 | 209,3 |
| TNF-alfa, DRG International | serum | 0,7 pg/ml | 6,6 | 4,5 | 488,4 |
| MMP-9 (human), DRG International | plasma | 0,05 ng/ml | 3,2-16,9 | 5,7-12,4 | 511,6 |
| ET-1; Human Big Endothelin-1 Enzyme, DRG International | serum | 0,23 pg/ml | 10,1-12,8 | 2,5-4,6 | 462,8 |
| PIIANP, DRG International | serum | 17,2 ng/ml | 3,4-6,6 | 4,8-7,4 | 348,8 |
| Human/mouse PDGF-AA Immunoassay, RnD Systems | serum | 2,29 pg/ml | 8,7 | 12,0 | 348,8 |
| NGAL, BioPorto Diagnostics | serum | 1,6 pg/ml | 2,8-4,8 | 4,4-4,6 | 511,6 |
| Human Ischemia Modified Albumina (IMA), USCN Life Science | serum | 3,3 ng/ml | 1,6-5,9 | 3,2-8,8 | 395,3 |
| NT-pro BNP (proBNP II) Roche Diagnostics | serum | 5 pg/mL (0.6 pmol/L) | 1,3-2,5 | 2,0-2,9 | 395,3 |
| hsCRP Roche Diagnostics | serum | 0.15 mg/L (1.43 nmol/L, 0.015 mg/dL) | 0,3-1,5 | 0,5-2,0 | 151,2 |
| SFLT-1, Roche Diagnostics | serum | 10 pg/mL | 1,3-1,9 | 2,3-4,3 | 395,3 |
| hsTnI, Roche Diagnostics | serum | 0.16 μg/L (ng/mL) | 2,7-10,0 | 4,7-13,4 | 279,1 |
| PLGF Roche Diagnostics | serum | 3 pg/mL | 1,0-3,3 | 1,5-5,1 | 430,2 |
| PAPP-A Roche Diagnostics | serum | 4 mIU/L | 1,1-2,0 | 1,4-2,8 | 146,5 |
| IL-6, Roche Diagnostics | serum | 1.5 pg/mL | 1,1-14,4 | 1,8-17,4 | 476,7 |
| VIT-D, Roche Diagnostics | serum | 3.00 ng/mL (7.50 nmol/L) | 2,2-6,8 | 3,4-10,8 | 488,4 |
| PCT, Roche Diagnostics | serum | 0.02 ng/mL | 1,1-7,1 | 1,6-8,7 | 395,3 |

ET-1= endothelin 1, hsCRP= high sensitivity C-reactive protein, hsTnI= high-sensitive Troponin I, IL-6= interleukin 6, IMA= ischemia modified albumin, MMP-9= matrix metallopeptidase 9, NGAL= neutrophil gelatinase-associated lipocalin, NT-proBNP= N-terminal pro b-type natriuretic peptide, PAPP-A= pregnancy-associated plasma protein A, PCT= procalcitonin, PDGFAA= platelet-derived growth factor, PIGF= placental growth factor, PIIANP= type IIA Collagen N-Propeptide, SFLT-1= soluble fms-like tyrosine kinase-1, TNF-alpha= tumor necrosis factor alpha, VIT-D= vitamin D

Table S2. Baseline characteristics at the hospital admission due to acute coronary syndrome.

| **Parametr** | | **N = 146**  **%/ mean + SD** |
| --- | --- | --- |
| Female (%) | | 41 |
| Age, years | Average | 59.6 ± 9.9 |
|  | < 50 | 17.1 |
|  | 50-65 | 50 |
|  | > 65 | 32.9 |
| BMI, kg/m^2^ | Average | 27.5 ± 3.8 |
|  | ≥ 25 | 71.9 |
|  | ≥ 30 | 21.2 |
| Clinical characteristics | Active smoking | 56 |
|  | Hypertension | 56.9 |
|  | Diabetes | 15.3 |
|  | Dyslipidaemia | 33.3 |
|  | COPD/Asthma | 2.8 |
|  | Previous myocardial infarction | 18.1 |
|  | Previous stroke | 3.5 |
|  | Previous PCI | 4.2 |
|  | Previous CABG | 4.2 |
|  | Killip class II or more | 5.6 |
| Type of ACS | UA | 11 |
|  | NSTEMI | 23 |
|  | STEMI | 66 |
| ACS risk assessment tool | GRACE inhospital prognosis (points) | 120.5 ± 25.5 |
|  | GRACE six-months prognosis (points) | 90.9 ± 22.5 |
|  | SIMPLE (points) | 21 ± 8.9 |
| Localization of coronary artery narrowings | LM | 0.8 |
|  | LAD | 33.1 |
|  | LCx | 8.6 |
|  | RCA | 50 |
|  | OM1 | 1.5 |
|  | OM2 | 1.5 |
|  | Descending aorta | 1.5 |
|  | Coronary artery bypass | 3.0 |
| Reperfusion therapy | POBA | 15.8 |
|  | PCI | 70.6 |
|  | Number of implanted stents | 1.3 ± 0.6 |
|  | TIMI before reperfusion | 0.7 ± 1.1 |
|  | TIMI after reperfusion | 2.8 ± 0.6 |
|  | CABG | 16.7 |
| Length of hospitalization (days) | | 10.5 ± 5.8 |
| Laboratory parameters | Maximum troponin I (ng/ml) | 57.2 ± 120.9 |
|  | Creatinine (mg/dl) | 0.94 ± 0.24 |
|  | MDRD (ml/min/1.73 m^2^) | 83.7 ± 21.1 |
|  | Total cholesterol (mg/dl) | 195.5 ± 45.2 |
|  | LDL (mg/dl) | 120.5 ± 38.9 |
|  | HDL (mg/dl) | 47 ± 12.7 |
|  | Triglicerides (mg/dl) | 151.5 ± 104.3 |
| TTE results | LVDD (mm) | 5.07 ± 0.47 |
|  | LVM (g) | 233.3 ± 64.2 |
| Pharmacotherapy at discharge | Antiplatelets | 90.4 |
|  | ACEI or ARB | 92.5 |
|  | Beta-blocker | 91.8 |
|  | Statins | 95.2 |
|  | All above | 80.8 |

ACEI= angiotensin-converting-enzyme inhibitors, ACS= acute coronary syndrome, ARB= angiotensin receptor blockers, BMI= body mass index, CABG= coronary artery bypass grafting, COPD= chronic obstructive pulmonary disease, HDL= high- density lipoprotein, LAD= left anterior descending coronary artery, LCx= the left circumflex coronary artery, LDL= low- density lipoprotein, LM= left main coronary artery, LVDD= left ventricular diastolic diameter, LVM= left ventricular mass, MDRD= Modification of **Diet** in Renal Disease, NSTEMI= non-STEMI= ST-elevated myocardial infraction, OM1= first obtuse marginal coronary artery, OM2= second obtuse marginal coronary artery, PCI= percutaneous coronary intervention, POBA= plain old ballon angioplasty, RCA= right coronary artery, STEMI= ST-elevated myocardial infraction, TTE= transthoracic echocardiography, UA= unstable angina

Normally distributed continuous variables are presented as mean ± SD.

Categorical variables are expressed as percentage
